# Colorectal cancer lymph node metastasis prediction with weakly supervised transformer-based multi-instance learning

**DOI:** 10.1007/s11517-023-02799-x

**Published:** 2023-02-21

**Authors:** Luxin Tan, Huan Li, Jinze Yu, Haoyi Zhou, Zhi Wang, Zhiyong Niu, Jianxin Li, Zhongwu Li

**Affiliations:** 1 Key Laboratory of Carcinogenesis and Translational Research (Ministry of Education), Department of Pathology, Peking University Cancer Hospital & Institute, Beijing, 100142 China; 2 Beijing Advanced Innovation Center for Big Data and Brain Computing, Beihang University, Beijing, 100191 China; 3 School of Computer Science and Engineering, Beihang University, Beijing, 100191 China; 4 Shenyuan Honors College, Beihang University, Beijing, 100191 China; 5 College of Software, Beihang University, Beijing, 100191 China; 6 Blot Info & Tech (Beijing) Co. Ltd, Beijing, China

**Keywords:** Colorectal cancer, Lymph node metastasis, Whole slide image, Multi-instance learning, Vision transformer, Deep learning, Computer-aided diagnosing

## Abstract

Lymph node metastasis examined by the resected lymph nodes is considered one of the most important prognostic factors for colorectal cancer (CRC). However, it requires careful and comprehensive inspection by expert pathologists. To relieve the pathologists’ burden and speed up the diagnostic process, in this paper, we develop a deep learning system with the binary positive/negative labels of the lymph nodes to solve the CRC lymph node classification task. The multi-instance learning (MIL) framework is adopted in our method to handle the whole slide images (WSIs) of gigapixels in size at once and get rid of the labor-intensive and time-consuming detailed annotations. First, a transformer-based MIL model, DT-DSMIL, is proposed in this paper based on the deformable transformer backbone and the dual-stream MIL (DSMIL) framework. The local-level image features are extracted and aggregated with the deformable transformer, and the global-level image features are obtained with the DSMIL aggregator. The final classification decision is made based on both the local and the global-level features. After the effectiveness of our proposed DT-DSMIL model is demonstrated by comparing its performance with its predecessors, a diagnostic system is developed to detect, crop, and finally identify the single lymph nodes within the slides based on the DT-DSMIL and the Faster R-CNN model. The developed diagnostic model is trained and tested on a clinically collected CRC lymph node metastasis dataset composed of 843 slides (864 metastasis lymph nodes and 1415 non-metastatic lymph nodes), achieving the accuracy of 95.3% and the area under the receiver operating characteristic curve (AUC) of 0.9762 (95% confidence interval [CI]: 0.9607–0.9891) for the single lymph node classification. As for the lymph nodes with micro-metastasis and macro-metastasis, our diagnostic system achieves the AUC of 0.9816 (95% CI: 0.9659–0.9935) and 0.9902 (95% CI: 0.9787–0.9983), respectively. Moreover, the system shows reliable diagnostic region localizing performance: the model can always identify the most likely metastases, no matter the model’s predictions or manual labels, showing great potential in avoiding false negatives and discovering incorrectly labeled slides in actual clinical use.

**Graphical Abstract:**

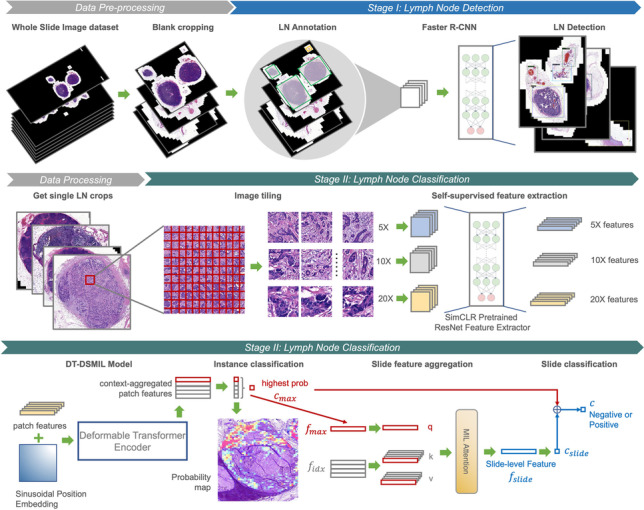

## Introduction

Colorectal cancer (CRC) accounts for approximately 10% of all cancer cases diagnosed and cancer-related deaths worldwide. CRC is also the third most common cancer in males and the second most common cancer in females [1]. The number of new CRC cases worldwide is estimated to increase to 2.5 million by 2035 [2]. Surgical resection of the tumor and associated regional lymph nodes remains the most effective treatment for CRC. Lymph node metastasis examined by the removed lymph nodes is considered one of the most important prognostic factors for the disease, which requires careful and comprehensive inspection by expert pathologists. The 7th and 8th editions of the American Joint Committee on Cancer have recommended the examination of at least 12 lymph nodes during surgical resection for CRC [3], which poses a significant challenge to the intensity and accuracy of the work for pathologists together with the increasing number of slides produced clinically.

Automated cancer detection in whole slide images (WSIs) has been a long-standing research area for decades since the traditional manual annotating procedure is time-consuming and error-prone. The application of deep learning to analyze WSIs is becoming increasingly important. Recent promising results in cancer diagnosis and detection, tumor micro-environment phenotype classification, and prognosis prediction reveal the great potential of WSI diagnostic methods based on deep learning [4–6]. These achievements can be attributed to the advances in computer vision and medical image analysis algorithms. However, these methods are still restricted by the high demand for a large-scale, thoroughly annotated dataset.

The typical paradigm of processing WSIs is the patch-wise processing method, which crops the gigapixel slide images into thousands of image patches with smaller dimensions, e.g., 224 × 224 pixels [7]. The patches are examined by a patch-wise classification network, e.g., a convolutional neural network (CNN), to obtain positive probability or to segment diagnostic regions within each patch, including tumor areas, stroma or smooth muscle, necrosis, and fibrosis [8]. The patch-wise results are further aggregated by simple aggregation methods like max or average pooling for slide-level tasks to obtain final global prediction results. The aforementioned patch-wise classification and global slide-wise aggregation procedure rely on patch-wise or even pixel-wise annotations, which are costly, time-consuming, and problematic due to the severely imbalanced data distribution between negative and positive patches and common and cancerous cells.

To address the issues mentioned above in patch-based fully supervised learning methods, some recent methods have studied WSI classification and segmentation systems in a weakly supervised manner that exploits slide-level diagnostic labels, which are readily available in the standard clinical systems or lymph-node level labels that can be collected relatively painlessly and achieved promising results [9, 10]. A typical solution for the weakly supervised WSI classification task in which only slide-level labels are available is the multi-instance learning (MIL) framework. In the MIL problem setting, if at least one patch with tumor cells is contained in the slide, the slide is labeled as positive. Some early works following the problem setting still exhibit the patch-wise classification manner but cast the slide-level labels to the patches with the highest probabilities instead of relying on the patch-wise labels [11, 12]. However, they still rely on the patch-level classifier to get the patch probabilities, and the final slide scores are acquired by simply averaging the patch scores with the same weights. Some other works train a small model to predict a weight for each patch score in the weighted averaging procedure or make the final decision on the patch predicted classes [13, 14].

Instead of averaging the patch scores to get the final slide predictions, some more recent works generate a feature vector for each patch and obtain a final global feature vector for the entire slide by aggregating the patch-level features. The global classification prediction is obtained by a classifier with the global-level feature instead of combining the patch-level prediction scores. Such works aggregate the patch-level scores with untrainable methods like concatenate [15, 16] and trainable methods like RNNs [17, 18], graphs [19, 20], and attention-based models [21–24]. The MIL-RNN model [17] casts the slide labels to the patches with the highest probability in the patch-level prediction and proposes to aggregate patch features with the highest probabilities with an RNN model. The DSMIL model [24] proposes to aggregate all patch features with the MIL attention mechanism. The CLAM model [25] aggregates the patch features with attention scores predicted for the patches, and an additional auxiliary clustering task is performed on the patches based on the ranking of the attention scores. And the most recent works directly apply the vision transformers, which are composed of procedures the same as the MIL framework like patching, feature extraction, feature aggregation, and decision-making to the WSI classification task [23]. With the MIL framework, only a label for the entire slide is required in the training phase, thus alleviating the heavy burden brought by the detailed annotations.

In addition to the benign and malignant classification as well as the genotyping of the entire slide as a whole [22, 26, 27], some previous works focus on other tasks like detecting DNA damage or mitotic figures with object detection methods, segmenting nuclei, glands, and different kinds of tissues with semantic segmentation methods and active learning [28–32].

In this study, we first develop a weakly supervised WSI classification model, DT-DSMIL, to identify metastasis in CRC lymph nodes with a transformer-based MIL model. In the DT-DSMIL model, the slides are first cropped into smaller patches, and their features are extracted by an ImageNet-pretrained ResNet-50 model. And then, the patch feature tokens are further processed and aggregated with each other with an encoder-only version of the deformable transformer model and the deformable attention mechanism. At the end of the model, a dual-stream MIL aggregator [24] is adopted to discover the critical patches within the slide and generate a global-level feature vector for the entire slide. The final slide-level classification results are made by a global-level classifier with the extracted global-level slide feature. DT-DSMIL is trained with slide-level labels only, while it is able to discover and illustrate the diagnostic regions and components with visualization maps.

Furthermore, based on the proposed DT-DSMIL model and the Faster R-CNN model, we developed a two-stage diagnostic system for the single lymph node classification task. The lymph nodes within the slides are detected and then cropped with the Faster R-CNN model in the first stage and then classified as negative or positive with the proposed DT-DSMIL model in the second. The lymph nodes within the slide are annotated with bounding boxes and corresponding binary positive/negative labels. The developed diagnostic system is trained with the annotations, and its performance for both tasks is evaluated. Furthermore, an extensive evaluation is performed on different subsets of the test dataset, including lymph nodes with or without neoadjuvant chemotherapy, lymph nodes with different histologic subtypes, and lymph nodes with different metastatic foci sizes. Visualized heatmaps are generated in the inference stage to interpret the prediction results and localize the tumor regions.

The main contributions of this paper can be summarized as follows:

1. We propose a novel and effective transformer-based MIL model, DT-DSMIL, for the CRC LN metastasis classification task. The DT-DSMIL can be trained with only the slide-level binary labels already available in the clinical medical record database to identify the WSIs and further localize the diagnostic regions. The performance of the proposed DT-DSMIL model is evaluated and compared with its predecessors.
2. Based on the proposed DT-DSMIL model, we develop a diagnostic system to detect, crop, and identify every single lymph node in the WSIs. Extensive artificial evaluations of the performance in identifying the majority types of metastases and localizing the diagnostic and tumor areas are carried out by expert pathologists on different subsets of the data.
3. We collected a clinical CRC LN metastasis dataset of 843 WSIs from 357 patients from 2019 to 2021. The dataset is annotated with slide-level binary positive/negative labels, bounding boxes, and binary labels for the lymph nodes in the slides. The test set is further annotated with dot annotations on the tumor areas and re-checked by two qualified pathologists blindly and independently. The proposed model and the developed diagnostic system are trained and evaluated with the dataset.

## Materials and methods

### Data collection

This study enrolled 843 digital slide images from 357 patients who underwent radical resection of primary CRC in Peking University Cancer Hospital between January 2019 and January 2021. Hematoxylin and eosin (H&E) stained sections are scanned with the Aperio AT2 digital pathology scanner (Leica Biosystems) with 40 × magnification and visualized with the Aperio ImageScope software. The corresponding complete clinical data and histopathology reports are collected for all slides. Slides with histological artifacts such as over fixation, poor staining, and bubbles are excluded, while slides with pen markers are adopted.

**Fig. 1:**
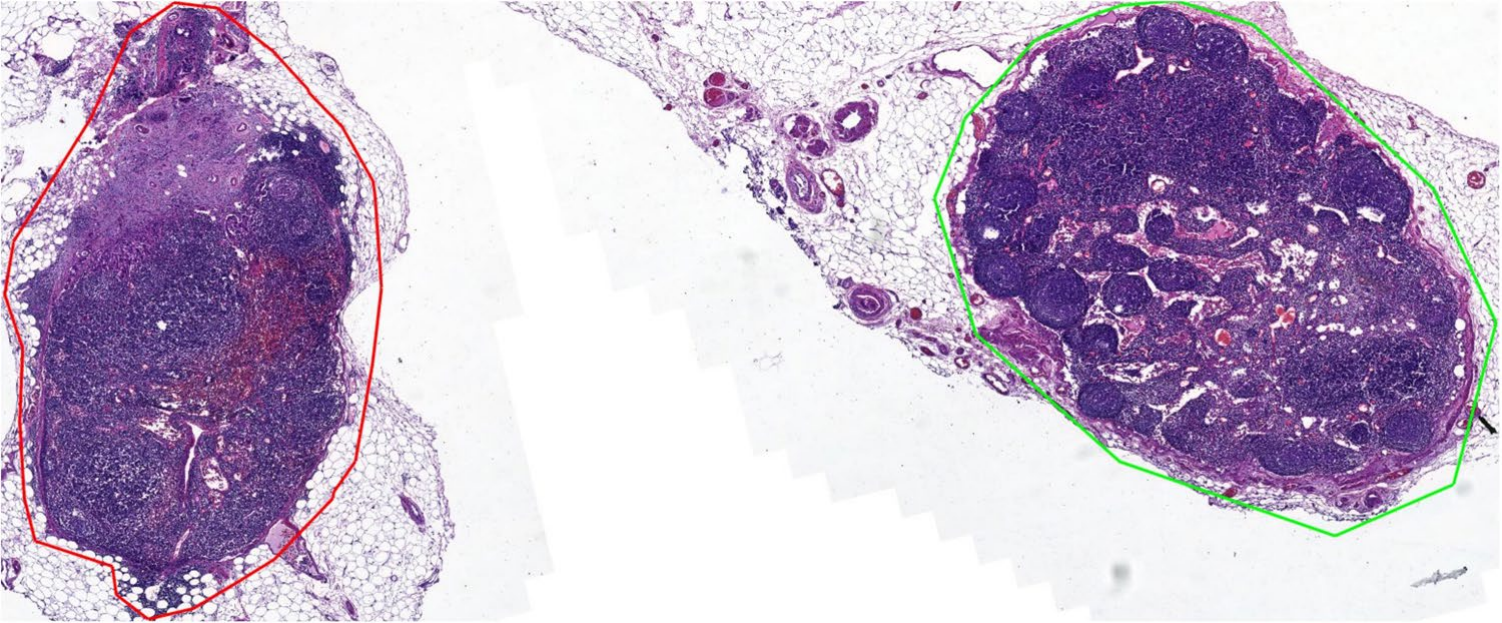
Examples of the collected slides in our dataset and their annotations

Among these 843 slides, 556 with positive lymph nodes are labeled positive, while the remaining 287 are labeled negative. For the slides labeled as positive, at least one positive lymph node (with metastasis) is contained, while for the slides labeled as negative, all the contained lymph nodes are negative and with no metastasis. Besides the binary labels, bounding boxes for lymph nodes and other isolated separatable tissues, such as tumor deposits, vessels, and fat within the slides, are collected for the single lymph node detection and classification task. Irregular structureless collections of lymphoid tissue with no fibrous capsule located in the fibro-adipose connective tissues are not counted as lymph nodes. Acellular mucin pools found in lymph nodes after neoadjuvant therapy are considered negative lymph nodes. Examples of the collected slides and their annotations, to be detailedly introduced later, are shown in Fig. 1.

For lymph node-level data composition, a total number of 2279 nodes are included. Eight hundred sixty-four (approximately 2/5) nodes are positive, and the remaining 1415 lymph nodes are negative. Irregular structureless collections of lymphoid tissue with no fibrous capsule located in the fibro adipose connective tissue are not counted as lymph nodes. Acellular mucin pools found in lymph nodes after neoadjuvant therapy are considered negative lymph nodes. The ground truth slide-level labels are determined by the slides’ clinical reports.

### Software and hardware requirements

All experiments are conducted on a high-performance computing cluster in the Beijing Advanced Innovation Centre for Big Data and Brain Computing. In particular, the experiments are conducted with one NVIDIA Tesla A100 PCI-E 40 GB GPU with the support of CUDA 11.3 and cuDNN 8.2 for GPU acceleration. PyTorch 1.11 is employed for model building and training, OpenSlide Python 1.12 for WSI file loading, and Python 3.8 for coding. Annotating for validating and testing is performed with the Automated Slide Analysis Platform (ASAP) with version 1.9.

### MIL-based slide classification with DT-DSMIL model

**Fig. 2:**
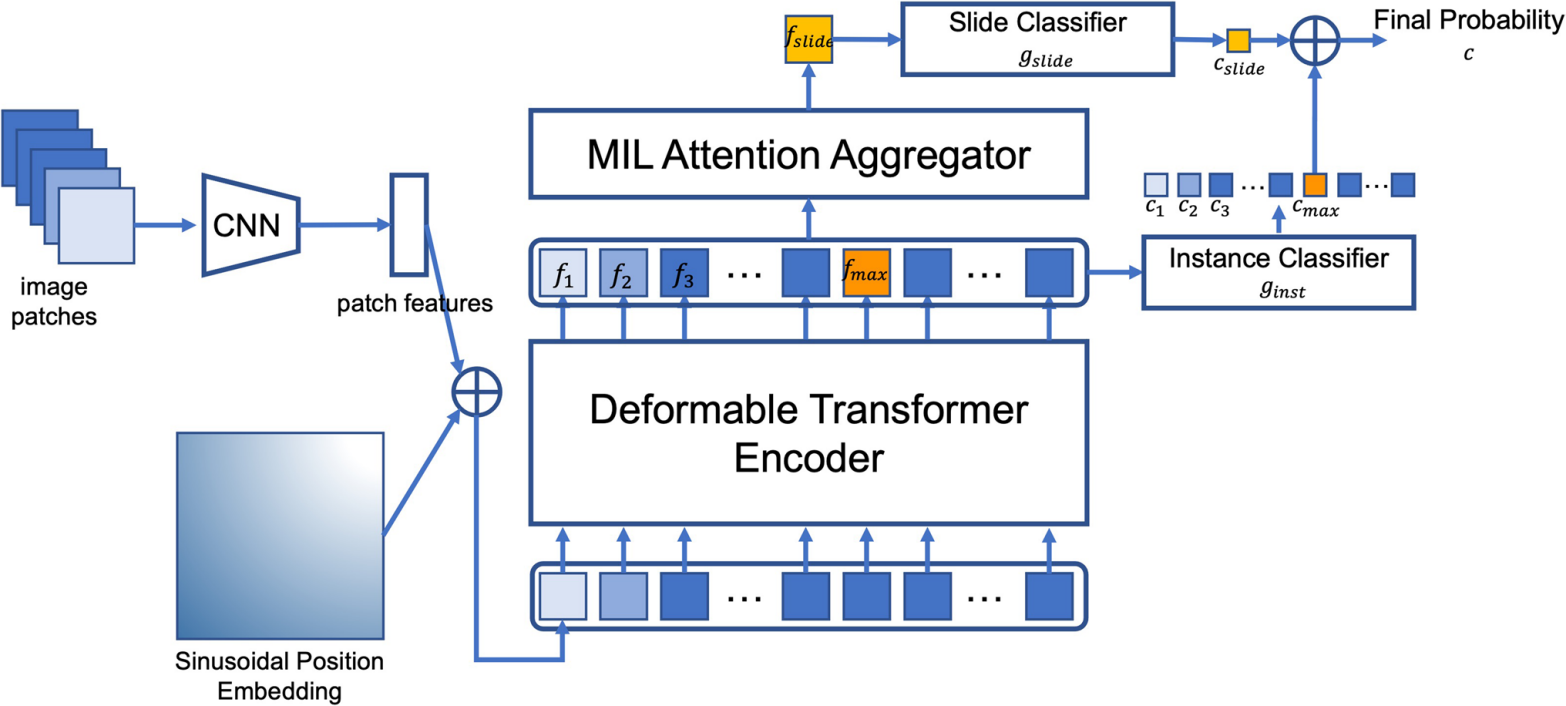
Diagram for the DT-DSMIL model, composed of a deformable transformer encoder and a dual-stream MIL attention head

The overall framework of the proposed DT-DSMIL model is illustrated in Fig. 2, which is composed of four components: a patch-wise feature extractor, a transformer-based local-level feature aggregator, a dual-stream MIL-based global-level feature aggregator to find out the most decisive patch within the entire slide and generate the global-level slide feature, and the final classifier to make the decision for each slide based on the feature for the critical patch and the feature for the entire slide.

In the preprocessing and the feature extraction phase, for each slide, the tissue regions are segmented by thresholding the saturation channel, and the regions are cropped into smaller patches with the same size as the ImageNet dataset ($256\times 256$ pixels) at the magnification of $10\times$ and with no overlapping. A 2048-d feature vector is generated for each patch with the patch-wise feature extractor implemented by an ImageNet pre-trained ResNet-50 model. The final classification layer of the model is removed, and thus the output of the modified ResNet-50 model is a feature vector with a length of 2048. Position embeddings are added to the patch features before the local-level feature aggregator. The simple sinusoidal absolute position embedding is adopted in our model. The position embedding vectors are the same length as the patch features (that is, 2048-d in our model). For the $idx$-th patch at position $({x}_{idx},{y}_{idx})$, its 2048-d position embedding is divided into two 1024-d vectors, for the *x*-axis and the *y*-axis separately. For each 1024-d vector at the position $pos$ in either axis, its value at the dimension of $2i$ or $2i+1$ is:

$$P{E}_{\left(pos, 2i\right)}=\mathrm{sin}\left(pos/{10000}^{2i/{d}_{\mathrm{model}}}\right)$$

$$P{E}_{\left(pos, 2i+1\right)}=\mathrm{cos}\left(pos/{10000}^{2i/{d}_{\mathrm{model}}}\right)$$

The two 1024-d position embeddings for the *x* or *y* dimension are concatenated, and a final 2048-d position embedding for both dimensions is acquired. A dimension-wise addition is performed between the position embeddings and the features.

The transformer-based local-level feature aggregator is implemented with an encoder-only version of the deformable transformer. The position-embedded local-level patch feature tokens are given to the transformer model as input, and the encoder of the transformer, which is composed of several stacked transformer blocks with multi-head deformable self-attention, feed-forward network, layer normalization, and GeLU activation function, aggregates the input feature tokens with each other based on their correlations. The output of the transformer encoder is the context-aggregated local-level patch feature tokens.

**Fig. 3:**
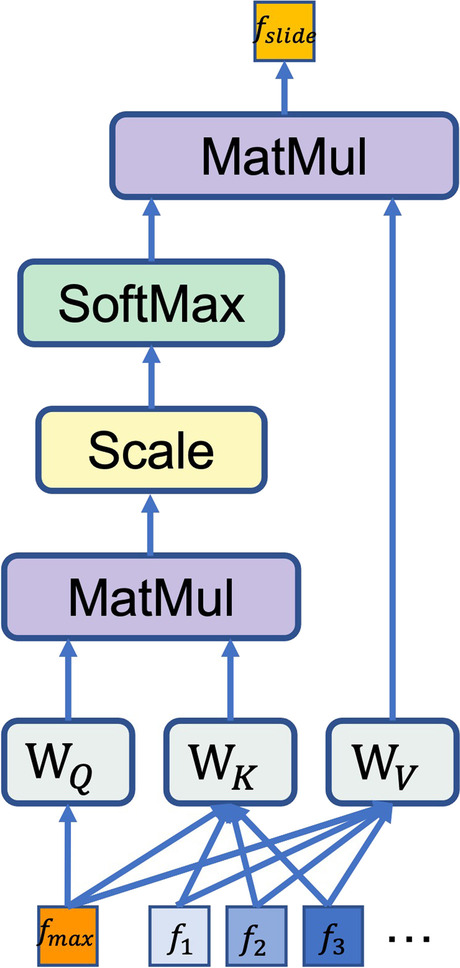
Diagram of the MIL attention mechanism in the DSMIL attention head, which can be viewed as a simplified version of the multi-head self-attention mechanism

Then, instead of the decoder part of the original transformer model, the dual-stream MIL aggregator is adopted to generate global-level slide features based on the most decisive patch within each slide. The most decisive patch is discovered with a local-level instance classifier ${g}_{inst}$ which predicts a score ${c}_{idx}={g}_{inst}\left({f}_{idx}\right)$ for the $idx$-th patch feature token, and the token feature ${f}_{\mathrm{max}}$ with maximum prediction score ${c}_{\mathrm{max}}$ is chosen as the most decisive patch feature token. Then the token ${f}_{\mathrm{max}}$ is aggregated with all the tokens by the MIL attention mechanism, which can be viewed as a simplified version of the original self-attention as shown in Fig. 3: only the attention scores between the selected token ${f}_{\mathrm{max}}$ and other tokens are computed, resulting one context aggregated global-level token ${f}_{\mathrm{slide}}$. Then a global-level classifier ${g}_{\mathrm{slide}}$ is applied to obtain a prediction score ${c}_{\mathrm{slide}}$ for ${f}_{\mathrm{slide}}$. Then the final prediction score $c$ for the entire slide is obtained by averaging the global-level prediction score and the local-level prediction score:

$$c=\frac{1}{2}\left({c}_{\mathrm{max}}+{c}_{\mathrm{slide}}\right)$$

### Metastases location with patch intensity and attention maps

Our developed diagnostic system is capable of locating diagnose-related decisive components within each slide with patch probability maps derived from the instance-level classifier and attention maps derived from the DSMIL aggregator.

The patch probability maps and the attention maps describe the prediction process from two aspects: the patch probability maps obtained by the instance-level classifier reveal the probability of each patch being positive in the local-level patch classification task. The patch within each image with the highest probability score is of the most importance and with the highest probability of containing tumor cells. However, the final loss value computation involves only one patch with the highest probability. Thus, the local-level prediction task training and its supervision signal are rather noisy. Therefore, the localization performance of the patch probability maps is unreliable, as shown in the artificial visualization result analysis. As the patch probability maps cannot sufficiently represent the entire slides, we introduce the attention scores generated by the global-level DSMIL aggregator, which represents the correlation between all other patches and the most decisive patch of each slide. The attention scores are extracted and visualized, resulting in an attention map for each slide. The higher attention weights mean that the corresponding patches are of higher importance in the global-level slide prediction. The attention maps describe the patches from a global view of a higher level. The attention scores for the patches reveal a higher connection with the tumor areas than the probability scores in the manual analysis. Furthermore, the visualized maps can serve as practical tools to avoid undetected positive and false negatives in actual clinical use, given the patch intensity and attention maps. The pathologists can easily double-check the model’s predictions by only reviewing the regions of the highest importance in the two maps.

### Diagnostic system for single lymph node detection, cropping, and classification

**Fig. 4:**
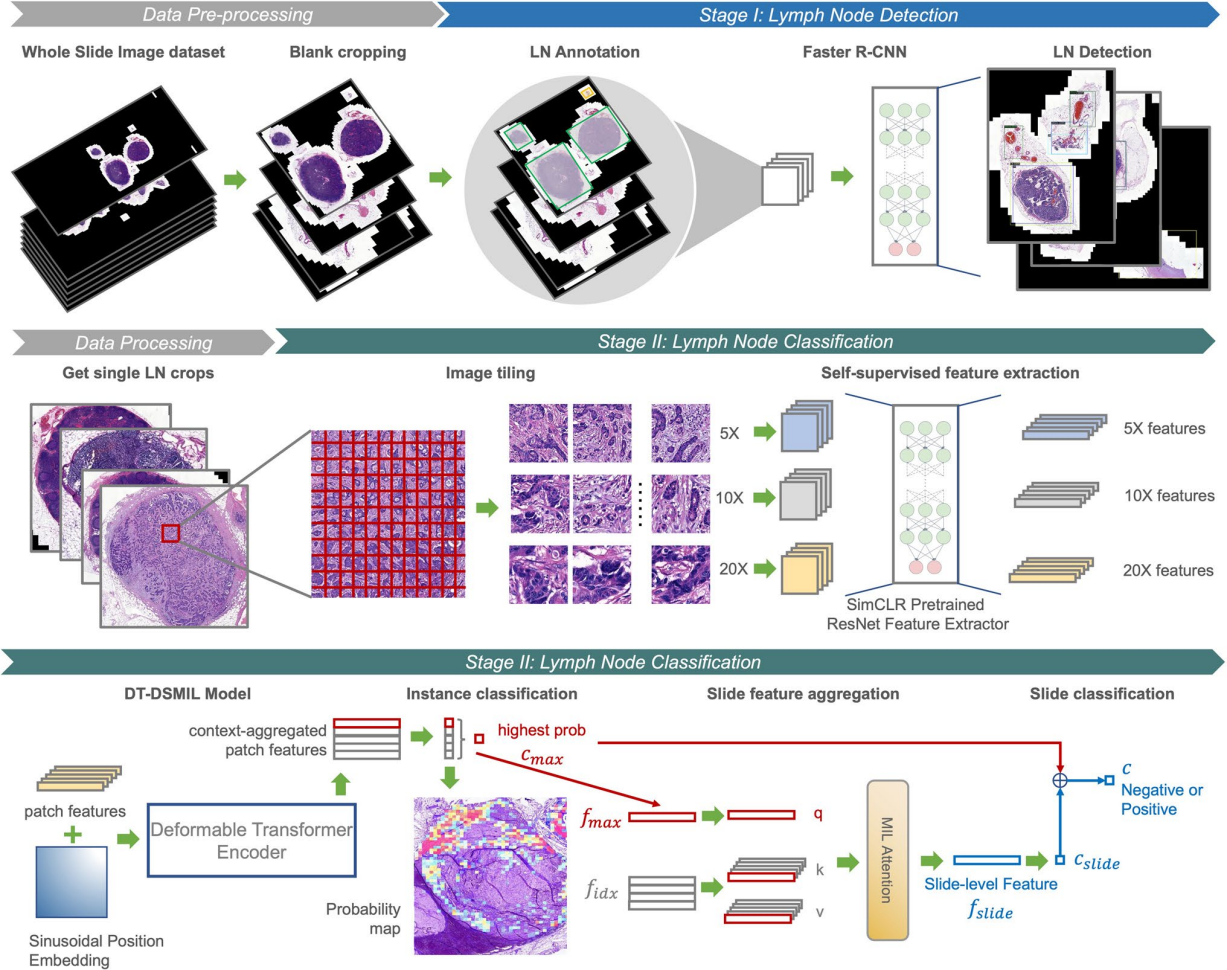
Diagram for the overall structure of the developed diagnostic system for single lymph node detection and classification, composed of data preprocessing, lymph node detection with a Faster R-CNN model, and classification of benign and malignant lymph nodes with a DT-DSMIL model

After proposing and evaluating the novel DT-DSMIL model, a diagnostic system for detecting, cropping, and classifying the single lymph nodes in the slides is developed based on the DT-DSMIL model and the Faster R-CNN model. The developed diagnostic system comprises two stages: one for distinguishing and localizing the lymph nodes against other tissues in the WSIs and another for classifying these lymph nodes as positive or negative. The overall architecture of the developed diagnostic system is illustrated in Fig. 4.

The first stage is accomplished with a lymph node detector based on Faster R-CNN [12]. Tissues within the WSIs are localized and classified as lymph nodes or other isolated tissues such as tumor deposits, vessels, and fat. Single lymph node images are cropped from the entire WSIs according to the model’s prediction. This stage is performed on WSIs at $5\times$ magnification to improve efficiency and reduce the computational cost, which is adequate for the pathologists to distinguish different tissue types.

The second stage is performed on the single-node images acquired in the first stage, where the output of the first stage, i.e., the lymph nodes, are further classified as positive or negative by applying the proposed DT-DSMIL model. The training and inference procedure is the same as for the entire slides, except that the input data is the cropped single lymph node images instead of the complete slides, and the image patching and feature extraction are conducted at the magnification of $20\times$ for higher accuracy. To make the diagnostic system more applicable in actual clinical use, the ResNet-50 patch feature aggregator is further pretrained by the SimCLR framework with the data in the training set after loading the ImageNet-pretrained weights to help the model get more information about the real-clinical data.

With the diagnostic system, not only the number of the lymph nodes within the slides but the benign and malignant of each lymph node can be obtained, which is consistent with the practice of clinically histopathological diagnosis.

### Statistics

The performance of the proposed WSI classification model is measured by accuracy, area under the receiver operating characteristic curve (AUROC, or AUC), precision, recall, and the F1 score. The 95% confidence intervals for AUC values are calculated with DeLong’s method. In our DT-DSMIL model, the final binary classifier predicts a probability score for each slide ranging from 0 to 1, which means the likelihood of the slide being positive, and the discriminative threshold is calculated and set according to the performance on the validation set. Probability scores higher than the above threshold are classified as positive, and scores lower than the threshold are classified as negative.

## Results

### Experimental settings

To evaluate the performance of our proposed DT-DSMIL model, experiments are conducted using the collected WSIs introduced in the Data Collection chapter. The WSIs are randomly split into training, validation, and testing sets with a ratio of 70:10:20, keeping the positive/negative class distribution and regardless of the patient’s information, resulting in 590, 84, and 169 slides in each dataset. The number of lymph nodes contained in the images in each dataset is 1598, 231, and 450, separately. After the dataset splitting phase, slides in the testing set are further verified, re-checked, and more detailed annotated with dots on the diagnostic regions for each positive lymph node by two qualified pathologists blindly and independently. As the testing set is treated separately and annotated in more detail, and an additional manual analysis procedure is conducted on the testing set introduced below, the cross-validation method is not used.

First, for the proposed DT-DSMIL model, the whole slide image classification task is performed: patch features are extracted for the entire slides, and the classification results for the whole of the slides are calculated. The performance metrics are obtained with the trained model, and a comparison is conducted between the DT-DSMIL model and its predecessors to demonstrate its effectiveness.

Then, for the proposed diagnostic system, the single lymph node detection and classification task is performed: the lymph nodes within the slides are detected first, and then, the classification result for each single lymph node is obtained in a similar way to the above. The performance metrics for the LN detection conducted by a Faster R-CNN model and the LN classification conducted by a proposed DT-DSMIL task are calculated and reported. Furthermore, more detailed performance analyses are performed. Specifically, we compare the DT-DSMIL model performance concerning the following aspects: performance with or without neoadjuvant chemotherapy; performance in different subtypes, including adenocarcinoma, mucinous carcinoma, and signet ring carcinoma; performance with different metastatic foci sizes. Moreover, the tumor morphologies relevant to the model’s decision-making are discovered with visualized probability maps and attention maps. Finally, artificial examination progress is conducted for the failure cases, especially the false negative ones. In such progress, we found out that our model is capable of localizing diagnostic regions even though the final prediction made by the model is false. Our model can help to reduce omitted positive nodes in manual annotations.

### Evaluation of the DT-DSMIL model

As introduced above, the DSMIL model and its predecessors (the DSMIL and the DT-MIL model) are trained and evaluated with the entire slides in the dataset, and performance metrics, including AUC, accuracy, precision, recall, and F1 score, are calculated. Results are shown in Table 1. The table shows that our proposed DT-DSMIL model outperforms its predecessorsn.

**Table 1:** Performance of our proposed DT-DSMIL and its predecessors. The highest value in each metric is marked in bold

Model	AUC	F1	Precision	Recall	Accuracy
DSMIL	0.9369	87.27	79.29	97.04	86.98
DTMIL	0.9743	93.58	89.35	**98.22**	91.24
DT-DSMIL (ours)	**0.9769**	**94.37**	**93.49**	95.27	**93.50**

### Evaluation of the diagnostic system

#### Numerical metrics of lymph node detection and classification

**Fig. 5:**
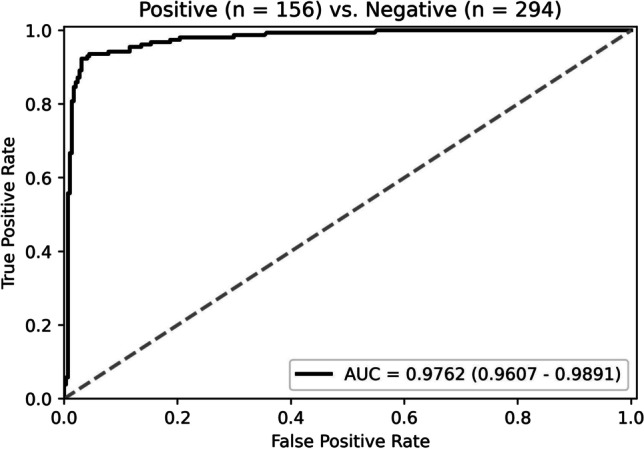
The ROC curve of our model in lymph node classification

To meet clinical requirements, a diagnostic system is developed to detect and further classify lymph nodes in the slides, and the system’s performance is evaluated on the testing set. The diagnostic system’s first stage, the Faster R-CNN model, is trained and evaluated with the entire slides. Its accuracy achieves 96.02%, obtained through manual analysis by experts of the test detection results. The trained Faster R-CNN model infers on the entire dataset, including the training, validation, and testing set. The obtained inference results, single lymph node images, are taken as the input of the diagnostic system’s second stage, which is a DT-DSMIL model. Detected lymph nodes are labeled as malignant or benign depending on whether they contain tumor cells, and non-lymph-node tissues misdetected as lymph nodes are labeled as benign. Performance metrics, including AUC, accuracy, precision, recall, and F1 score for the second-stage DT-DSMIL model, are obtained by training and evaluating using the single lymph node images. The model performs well for metastatic foci in lymph nodes, with an AUC of 0.9762 (95% confidence interval [CI]: 0.9607–0.9891) and an accuracy of 95.33%, and the ROC curve is shown in Fig. 5. The precision of the single lymph node classification is 0.9411, the recall is 0.9231, and the F1 score is 0.9320.

#### Model performance on cases with or without neoadjuvant chemotherapy

**Fig. 6:**
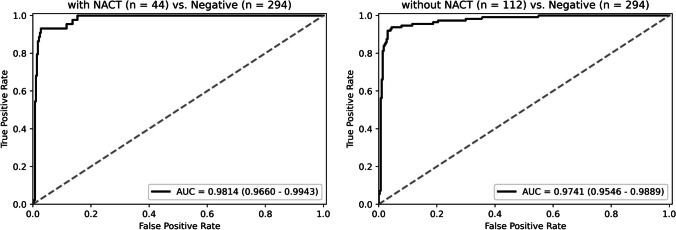
The ROC curve of our model on cases with or without neoadjuvant chemotherapy

Among the 450 lymph nodes in the test set, 44 are from 32 patients with neoadjuvant chemotherapy (NACT). These lymph nodes produce a post-treatment response, such as metastatic tumor shrinkage, mucin pools, fibrosis, or foamy histiocytes, but the residual tumor can still be seen in all of them. The ROC curves of our model on cases with or without NACT are shown in Fig. 6. The model performs well in both, with an AUC of 0.9814 (95% CI: 0.9660–0.9943) and 0.9741 (95% CI: 0.9546–0.9889), respectively. And the accuracy of cases with or without NACT is 91.9% and 93.2%.

#### Model performance in different histologic subtypes

**Fig. 7:**
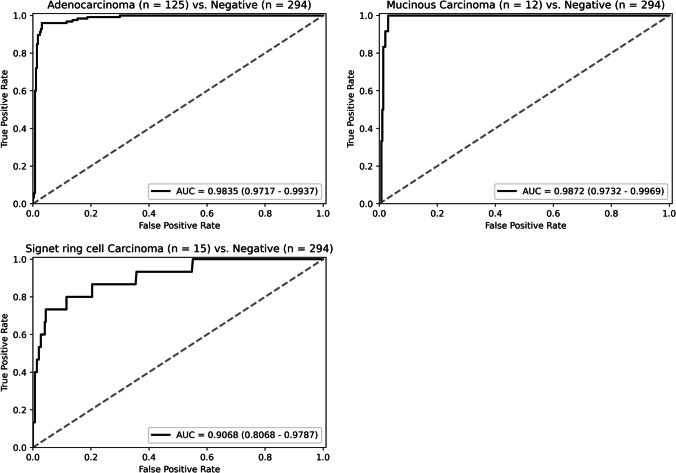
The ROC curve of our model in different histologic subtypes

The positive lymph nodes in the test set are divided into three groups, the adenocarcinoma, the mucinous carcinoma, and the signet ring carcinoma, according to different histological subtypes of the metastatic lesions, and the ROC curves of each group are shown in Fig. 7. Our DT-DSMIL model performs well on the adenocarcinoma and the mucinous carcinoma group, with the AUC of 0.9835 (95% CI: 0.9717–0.9937) and 0.9872 (95% CI: 0.9732–0.9969) and the accuracy of 96.0% and 100%, respectively. In contrast, the performance is relatively poor in the signet ring carcinoma group, with an AUC of 0.9068 ((95% CI: 0.8068–0.9787) and an accuracy of 60.0%. In addition, there are four lymph nodes with micropapillary adenocarcinoma, three correctly identified, while the remaining one with a maximum diameter of less than 0.2 mm is omitted.

#### Model performance regarding metastatic foci size

**Fig. 8:**
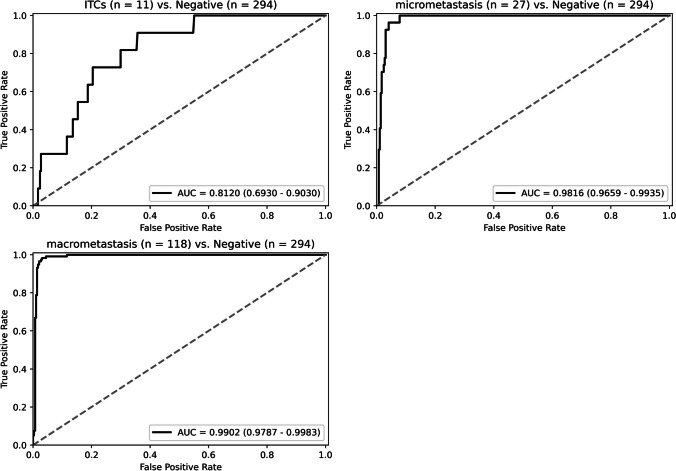
The ROC curve of our model regarding metastatic foci size

According to the CAP Cancer Reporting Protocols (Colon and Rectum, Resection, version 4.2.0.1) [33], isolated tumor cells (ITCs) are defined as single tumor cells or small clusters of tumor cells measuring less than 0.2 mm. Metastatic deposits with the size of 0.2–2.0 mm are called micro-metastasis, and deposits larger than 2.0 mm are called macro-metastasis. The ROC curves of our model in identifying lesions of different sizes are shown in Fig. 8. Our model performs well for micro-metastasis and macro-metastasis, with the AUC of 0.9816 (95% CI: 0.9659–0.9935) and 0.9902 (95% CI: 0.9787–0.9983) and the accuracy of 92.6% and 98.3%, respectively. In contrast, the performance is relatively poor in ITCs, with an AUC of 0.8120 (95% CI: 0.6930–0.9030) and an accuracy of 27.3%.

### Diagnostic morphology in lymph node classification

**Fig. 9:**
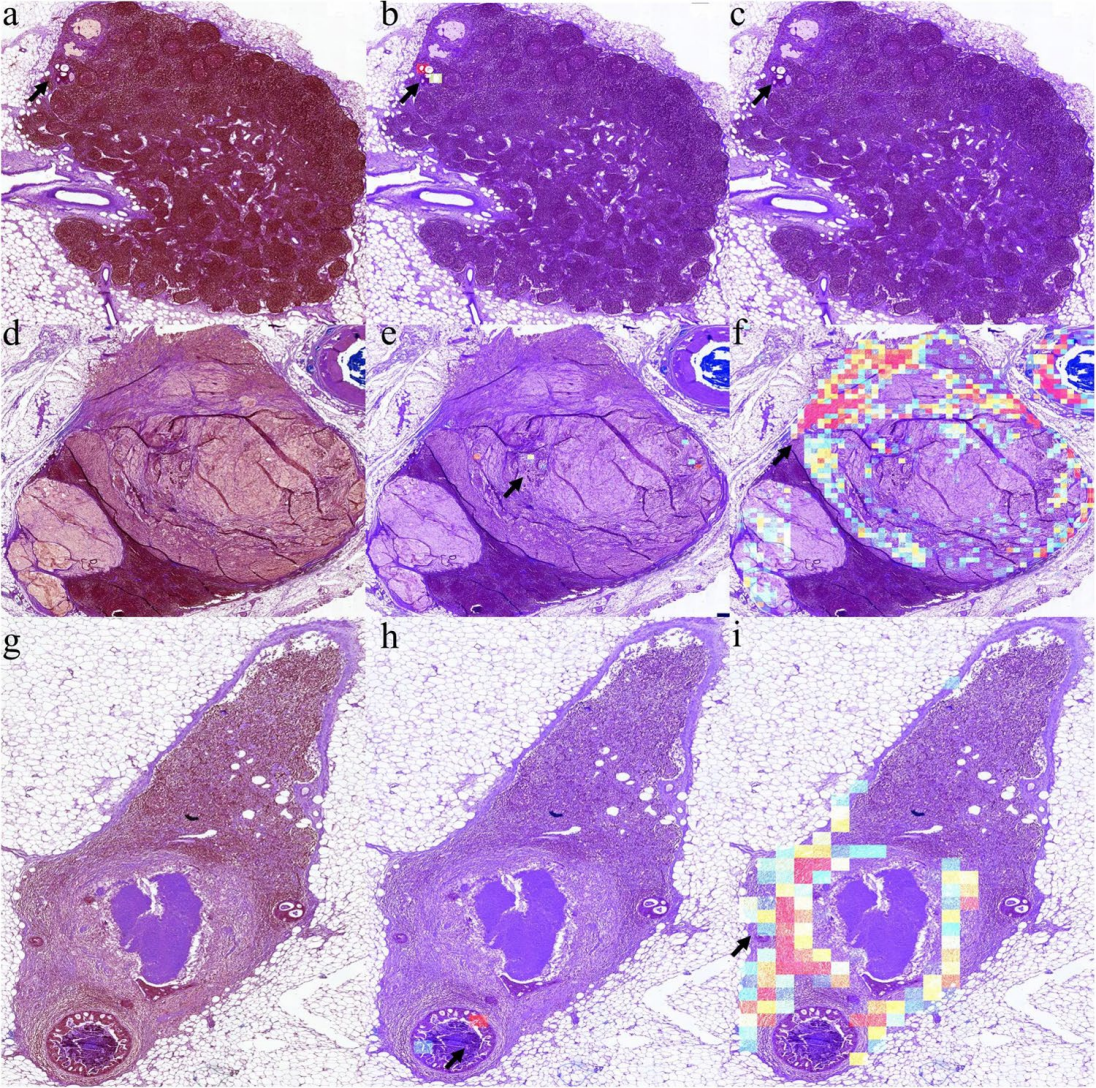
Examples of visualization images for lymph node classification task. In each row, the first figure is a color-processed lymph node image (**a**, **d**, and **g**), the second (**b**, **e**, and **h**), and the third (**c**, **f**, and **i**) is an attention map and a probability map corresponding to the lymph node image. **a**–**c** An example of ITCs shows that tumors with typical glandular structures could be identified. **d**–**f** An example of mucinous carcinoma with signet ring carcinoma shows that our model is poor at identifying tumors with scattered or solid structures in the attention maps. **g**–**i** An example of a post-treatment lymph node shows that fibers are more evident than tumors in the probability maps

Our model is able to interpret its classification result with the visualization heatmaps, including the patch-wise classification probability maps and the attention maps. As shown in Fig. 9, the tumor morphologies related to the prediction are highlighted. Both maps are assessed by the pathologists, and we find out that the attention maps, which reveal the connection and the importance of each patch in the global-level feature extraction and decision-making, are more closely associated with the location of the lesions and thus are more vital. In the attention maps, tumor cells are particularly distinguishable. In identifying adenocarcinoma, our model is more sensitive to glandular, cribriform, and papillary structures. Even in lymph nodes with ITCs, tumors with typical glandular structures can be clearly identified (Fig. 9a–c). In identifying mucinous and signet ring carcinoma, our model is relatively poor at identifying tumors with scattered or solid structures. Only if tumor cells form a cord or papillary shape can be identified by our model (Fig. 9d–f). As for the probability maps, fibers are more evident than tumors, especially in lymph nodes after NACT (Fig. 9g–i). The poor performance of the probability maps generated by the local-level patch-wise classification might be caused by the noisy supervision signals in the training scheme. Therefore, attention maps should be chosen as the significant visualization tool in our model.

### Error case analysis

Among the 450 single-lymph node images in the test set, twelve are predicted to be false-negative, and nine are false-positive. Among the false-negative lymph nodes predicted, eight are ITCs with adenocarcinoma and signet ring carcinoma, two are micro-metastasis with signet ring carcinoma and micropapillary adenocarcinoma, and the remaining two are signet ring carcinoma macro-metastasis. Among the false-positive lymph nodes predicted, four delineated lymph nodes have tumor deposits outside, two are identified as the fibrous capsule of the lymph nodes by the pathologists, one is a deformed lymphocyte mass, and the last one is a lymphatic sinus.

In our artificial failure case evaluation and verification progress, we found that a positive lymph node with adenocarcinoma micro-metastasis is mislabeled as negative, and the model is able to identify it correctly. The metastatic site of this lymph node has a typical glandular structure, but the tumor cells are slightly deformed due to the production.

## Discussion

The presence of metastasis in lymph nodes is a critical prognostic indicator for patients with CRC and an essential determinant of clinical decision-making [34]. To meet the accuracy requirement in the histopathologic diagnosis of tumors and reduce the increasing burden on the pathologist, the application of machine learning, particularly deep learning, has been seen as a milestone for the healthcare sector in the next decade [35].

In this paper, we propose a new MIL-based WSI diagnostic model, DT-DSMIL, for CRC lymph node metastasis classification and develop a two-stage diagnostic system to distinguish different single lymph nodes in the WSIs. Our model only requires the positive/negative labels for entire slides or single lymph nodes while able to identify the critical and diagnostic regions and thus substantially reduces the requirement of detailed annotations and reduces experts’ heavy burden of careful patch-wise or even pixel-wise annotation compared with previous computational histopathology diagnostic systems.

Our model performs well in classification tasks on all tumor subtypes except for the identification of ITCs. Although previous works have proposed different algorithms with different advantages, identifying ITCs in lymph nodes is still one of the most challenging points in developing computer-aided methods for identifying lymph node metastases. In the study of Chuang et al., their model’s performance of detecting ITCs in lymph nodes of CRC achieves an AUC of 0.7844 in a single-lymph-node-level test set with five samples [36]. However, the predictive accuracy of micro-metastasis and macro-metastasis may be more clinically significant than that of ITCs. In clinical practice, ITCs are recorded as N0, and the number of lymph nodes is presented separately in the pathology reports [37, 38]. The nodes indeed considered eroded by cancer are those eroded by micro-metastasis and macro-metastasis [39].

The misclassified positive lymph nodes, or the false negatives, can be divided into two categories through artificial analysis of the incorrect predictions: small clusters of adenocarcinoma metastasis without clear glandular structure and signet ring carcinoma metastasis. What they have in common is that they have no apparent morphological features, which are not the characteristics of tumor cells but a feature of how the tumor cells cluster. Our model is more sensitive to glandular, cribriform, and papillary structures but not scattered or diffuse distribution. This pattern has been found in other previous studies as well. For example, in the study of Hu et al., the false-negative rate in signet ring cells is 6.67%, and in poorly differentiated adenocarcinoma is 15.11% [40]. The sensitivity of our system to adenocarcinoma structures is also reflected in tumor deposits [41]. In the absence of lymph node metastasis, tumor deposits are recorded as N1c. In our model, four of the nine false-positive lymph nodes are due to the identification of tumor deposits.

In addition to classifying the lymph nodes, our model can effectively identify tumor-related diagnostic components in the positive lymph nodes with the DSMIL attention head. Two visualization maps, including the probability map generated by the patch-level classifier and the attention map generated by the global-level feature aggregator, are developed to explain the model’s predictions and to realize local-level patch predictions. And as previously described, the attention maps are more informative and of higher importance. However, from the visualization maps, we can find out that the final predictions are determined by patches with metastasis and patches with fibrosis. The probability map always highlights part of fibrosis in addition to the tumor cells. This may explain why our model performs better in cases with NACT than without NACT.

## Limitations and future work

Though our proposed DT-DSMIL significantly outperforms its predecessors in most performance metrics by a large margin, its recall deteriorates, and recall is a more concerned score in pathological diagnosis. To compensate for this, we choose a higher magnification, $20\times$ for the single lymph node classification task, instead of the magnification of $10\times$ for the whole slide classification. However, the backbone of our DT-DSMIL model, the deformable transformer, keeps the spatial dimensions in the input, which results in memory inefficiency, especially when faced with sparse slides, and limits the choice of maximum magnification. For example, in cases where multiple lymph nodes are placed on the same slide but far apart, the blanks must be kept in the input feature map. Thus, one possible direction is dealing with the input slides’ sparsity.

Besides, though our model only requires the binary labels for the slides to accomplish slide-level classification, the bounding boxes and binary labels for each lymph node are still needed in developing the diagnostic system for detecting and classifying single lymph nodes. Having witnessed the recent progress in weakly supervised object detection in natural images, we believe it is possible to accomplish such single lymph node-level tasks with a total number of all lymph nodes and malignant lymph nodes among them. And recent progress in semi-supervised learning and domain adaptive learning can help introduce more data from different data sources to improve the model’s performance [42]. Therefore, they are also the directions of our follow-up research.

Furthermore, it is acceptable in clinical practice to achieve nearly 100% accuracy and accurate localization performance with a small number of detailed annotations, such as the dot annotations collected in our study but only used in the manual result analysis stage, not the model development. Thus, weakly supervised methods that can achieve higher performance with few annotations and methods like human-in-the-loop and active learning models that can progressively collect annotations for the most critical and informative samples can be of great potential.

## Conclusion

In this paper, we first developed a weakly supervised WSI classification model, DT-DSMIL, based on the transformer model and the multi-instance learning framework to identify metastasis in CRC lymph nodes with merely slide-level diagnostic labels instead of detailed annotations. Then, a diagnostic system for detecting and further classifying each single lymph node in the slides is developed with a Faster R-CNN object detector and the proposed DT-DSMIL model. Though such coarse-grained annotations do not provide spatial information on the metastasis within lymph nodes, our model can still find the most diagnose-related components. The proposed model and the developed system are able to solve actual problems in clinical practice, and future work can be done to improve performance further.

## Data Availability

The datasets used and analyzed during the current study are available from the corresponding author upon reasonable request.
